# Obituary for Prof. Helena Rašková, MD., DSc., Dr.h.c. ^*^2 January, 1913 ^†^13 April, 2010

**DOI:** 10.2478/v10102-010-0008-0

**Published:** 2010-06

**Authors:** Jana Navarová, Eduard Ujházy

**Affiliations:** 1 Slovak Toxicology Society SETOX, SK-84104 Bratislava, Slovakia; 2 Institute of Experimental Pharmacology & Toxicology, Slovak Academy of Sciences, SK-84104 Bratislava, Slovakia

*When I die, nothing will happen and change in the world,*

*just a few hearts will shiver, in the dew like flowers in the morning…*

J. WOLKER

Professor MUDr. Helena Rašková, DrSc., nestor of Czech and Slovak pharmacology, passed away on April 13, 2010, at the age of 97 years.

The entire life of Professor Rašková was bound to Slovakia. As she put it herself, one half of her heart belonged to the Czech lands, while the other half belonged to Slovakia.

As early as in 1946, she established contacts with Professor František Švec, Head of the Institute of Pharmacology at the School of Medicine, Comenius University in Bratislava, and since that time, until her last days, she had been in continuous touch with her Slovak colleagues, among them largely pharmacologists and toxicologists.

In 1959 she succeeded in separating pharmacology and physiology as two separate branches of science, and founded the Czechoslovak Society of Pharmacologists, becoming President of the Society.

Professor Rašková founded the Institute of Experimental Pharmacology of the Slovak Academy of Sciences as a separate unit within the Slovak and Czech Academies of Sciences, which enabled a whole generation of Slovak scientists to travel abroad to the developed world. She took an active part in the education of Slovak pharmacologists, and she supported the development pharmacology, actively helping young Slovak pharmacologists obtain postgraduate education at renowned European and overseas pharmacological centres during the totalitarian era in Czechoslovakia.

Professor Rašková introduced to the scientific community two generations of Slovak pharmacologists from academic as well as university centres. Of the 18 university professors whom she taught and educated, 11 came from Slovakia. They have been working at Czech, Slovak, German and overseas universities.

Following the split of Czechoslovakia in 1993, Prof. Rašková initiated the organization of periodical conferences in toxicology that have been organised alternating in the Czech Republic and in Slovakia. Despite her advanced age, she actively participated in each of these conferences and, due to her immense professional credit, managed to ensure the participation of leading lecturers from abroad. This contributed greatly to improving the niveau of the conferences along with the interest of other countries in Slovak toxicology within its integration into the European Union.

Besides this, Professor Rašková devoted considerable effort to the development and improvement of professional as well as methodological work of Slovak pharmacological centres, contributing significantly with her experience and broad-sightedness. She always highly appreciated the friendship and support of her Slovak colleagues, pointing out that Slovakia has always turned a friendly face to her in times that were rather troubled for her, and appreciated the fact that we did not fear to invite her repeatedly to Slovakia and acknowledge her work in an era when in the Czech part of Czechoslovakia this was not possible. She would say that this-however not only this-is why Slovak pharmacology and toxicology will be to her a heart matter forever.

The scientific activities of Professor Helena Rašková are vast and many-sided. She wrote more that 500 original scientific papers and several monographs.

She published the results of her research into anaesthetics, curare-like substances, anti-thyroidal compounds, analeptics, hypnotics and other pharmaceutics in renowned international scientific journals.

Her original complex of publications on the pharmacology of bacterial toxins and their effect on the outcome of drugs in the body, as well as her contributions to the non-specific resistance of the organism was particularly highly esteemed also internationally.

She published her systematic results of research into anti-metabolites (6-azauracil, 6-azauridine), active substances of plant origin (chamazulene, bisabolol), and took part in clinical trials of the above preparations as well as of anti-metabolites.

It is remarkable that Professor Rašková did not stop her scientific work even when she was forced to work in agriculture. Her research from this period resulted in internationally respected achievements. By introducing anti-infectious conditions into cattle breeding, she contributed to the management of massive infections in diarrhoea-like diseases by elaborating an original method of oral re-hydration.

Her experience with pharmacological intervention in animal stress led to the recommendations in human medicine not to separate the mother and the neonate postpartum but leave them together. Later even the WHO integrated her suggestions into their agenda.

Besides her scientific work, Professor Rašková made a considerable contribution in building up the Czechoslovak Society of Pharmacology, of which she was President for many years, and it was her credit that many important international events took place. One of them was the 2^nd^ International Congress of Pharmacology in Prague in 1963, at which Professor Rašková was appointed Congress Chairman.

In 1961, together with Professor Uvnäs from Sweden, Professor Rašková established an independent international society of pharmacology, IUPHAR, of which she was an honorary member until her last days.

Indisputable are the merits of Professor Rašková in the history of drug toxicology and the formation of the European Society for the Study of Drug Toxicity, a forerunner of the present-day European Toxicological Society EUROTOX.

Professor Rašková had been a member, and after her retirement, an honorary member of a number of scientific societies and editorial boards in Slovakia as well as abroad.

To provide a list of all decorations and honours given to Professor Rašková would take a lot of space. Of them, let us mention only that she had been decorated with the Finnish Sibelius Medal, the French medal of the of Académie de Lutec, the Gold Medal of European Pharmacological Societies for her contributions to the development of world pharmacology, as well as all the decorations obtained from the Slovak Academy of Sciences: Gold Medal of the Slovak Academy of Sciences, Honourable Gold Medal of the Slovak Academy of Sciences for her contributions to biological sciences, and the Memorable Medal of the Slovak Academy of Sciences. Professor Rašková was an honorary member of the Learned Society of the Slovak Academy of Sciences.

Dear Professor, we will always remember you with affection and respect. We all miss you very much.

On behalf of your colleagues and friends,

*Jana and Edo*

**Figure:**
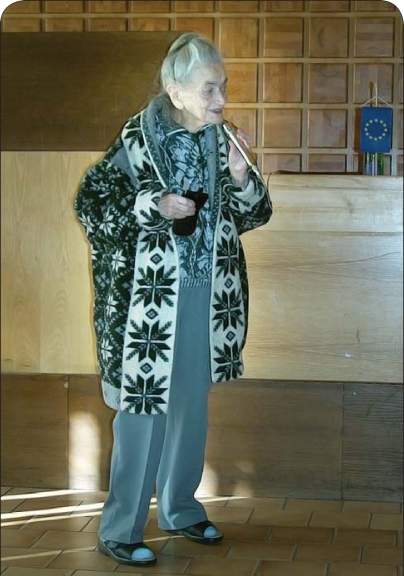
Professor Rašková at the TOXCON conference (Slovakia, 2004)-

